# Supplementary material for: Hypoxia-enhanced Blood-Brain Barrier Chip recapitulates human barrier function and shuttling of drugs and antibodies
Source: Nat Commun. 2019 Jun 13;10:2621. doi: 10.1038/s41467-019-10588-0 (PMC6565686; doi:10.1038/s41467-019-10588-0)
Supplement: Supplementary file 1 — Supplementary Information [file 41467_2019_10588_MOESM1_ESM.pdf]

Supplementary Information

Hypoxia-enhanced Blood-Brain Barrier Chip recapitulates human barrier function and shuttling of drugs and antibodies

Park et al.

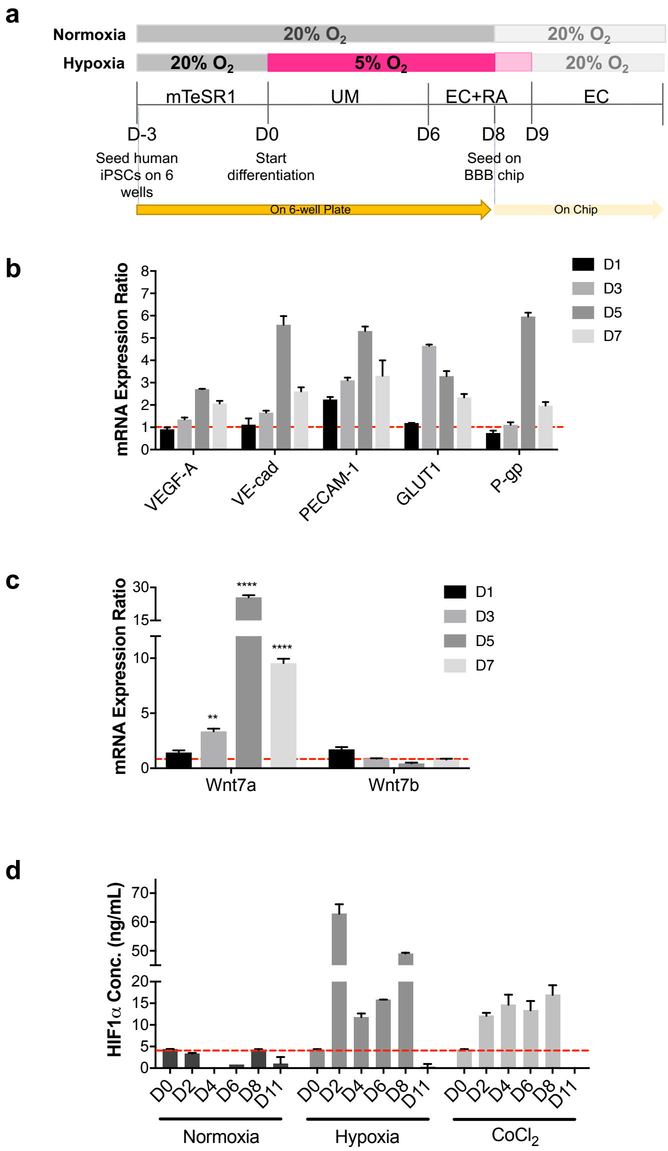

Supplementary Figure 1

(a) Timeline for the differentiation of the iPS cells to the human BMVECs, and seeding on the BBB chips. **(b)** Fold changes of mRNA expressions of VEGF-A, VE-cadherin, PECAM-1, GLUT1, and P-gp during differentiation (D1-D7) of iPS-hBMVECs under hypoxia relative to normoxia analyzed by qRT-PCR. **(c)** Relative fold change in mRNA expression of Wnt7a and Wnt7b in iPSC differentiated under hypoxic condition compared to normoxia during the

differentiation process (D1-D7). **(d)** ELISA analysis for HIF1α protein expression during differentiation of iPS-BMVEC (D0-D8) and after differentiation (D11).

Supplementary Table 1: Delta Ct values that used for generating Supplementary Fig. S1b

	Delta Ct							
**Hypoxia**	**D1**		**D3**		**D5**		**D7**	
**VEGF-A**	6.733	6.475	5.314	5.212	4.894	4.701	5.361	5.594
**VE-cad**	10.121	9.365	9.752	9.602	9.151	8.950	8.750	8.630
**PECAM-1**	13.104	13.246	13.064	12.947	12.691	12.584	12.408	12.164
**GLUT1**	4.618	4.592	4.020	3.985	3.265	3.070	1.760	1.831
**P-gp**	7.902	7.471	6.943	7.234	4.609	4.693	6.184	6.127
**Normoxia**	**D1**		**D3**		**D5**		**D7**	
**VEGF-A**	7.219	5.679	5.554	5.822	5.867	6.592	5.879	7.408
**VE-cad**	10.855	8.828	2.440	18.354	10.612	12.449	9.355	10.900
**PECAM-1**	14.808	13.865	13.146	16.125	10.929	19.165	14.139	14.366
**GLUT1**	4.475	5.217	6.099	6.336	4.800	4.968	2.373	3.720
**P-gp**	7.098	7.355	7.169	7.284	6.717	7.736	6.438	8.015

Supplementary Table 2: Delta Ct values that used for generating Supplementary Fig. S1c

	Delta Ct							
**Hypoxia**	**D1**		**D3**		**D5**		**D7**	
**Wnt7a**	13.69256	13.30081	12.82108	13.03322	11.95168	11.84094	10.56691	10.61591
**Wnt7b**	14.05247	14.4123	14.12736	14.05446	14.29699	13.8221	14.07046	14.01229
**Normoxia**	**D1**		**D3**		**D5**		**D7**	
**Wnt7a**	14.12309	13.87691	14.39285	14.9408	16.19843	16.93883	13.69824	14.05739
**Wnt7b**	15.09148	14.90852	13.87257	13.97206	12.45698	13.30491	14.12939	13.96345

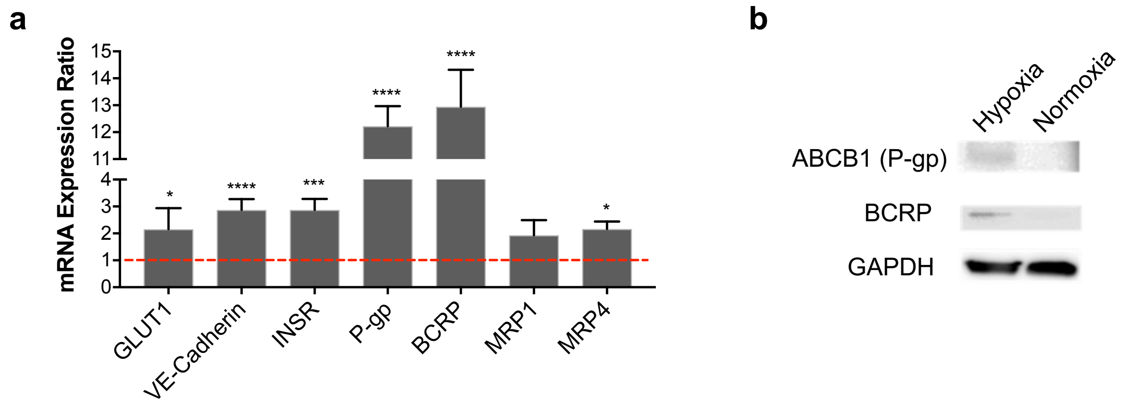

Supplementary Figure 2

(a) Relative fold changes in mRNA expressions of GLUT1, VE-cadherin, insulin receptor (INSR), P-gp, BCRP, MRP1, and MRP4 in iPS-BMVEC differentiated under hypoxic versus normoxic conditions. **(b)** P-gp and BCRP protein expression of the iPS-BMVEC differentiated under hypoxia and normoxia were compared using a Western Blot. GAPDH was used as a control protein.

Supplementary Table 3: Delta Ct values that used for generating the Supplementary Fig. S2

	Delta Ct							
	Normoxia				Hypoxia			
**GLUT1**	2.818	2.975	2.944	3.051	2.403	2.354	1.439	1.450
**VE-Cadherin**	9.181	8.716	8.808	9.142	7.490	7.192	7.386	7.669
**INSR**	7.049	6.715	6.758	7.174	5.538	5.483	5.171	
**P-gp**	7.911	7.649	7.983	7.776	4.267	4.265	4.239	4.088
**BCRP**	8.333	7.994	8.635	8.162	4.373	4.546	4.687	4.687
**MRP1**	9.684	9.456	9.822	9.575	9.165	9.023	8.363	8.350
**MRP4**	9.052	8.840	9.072	8.909	7.919	8.065	7.620	7.810

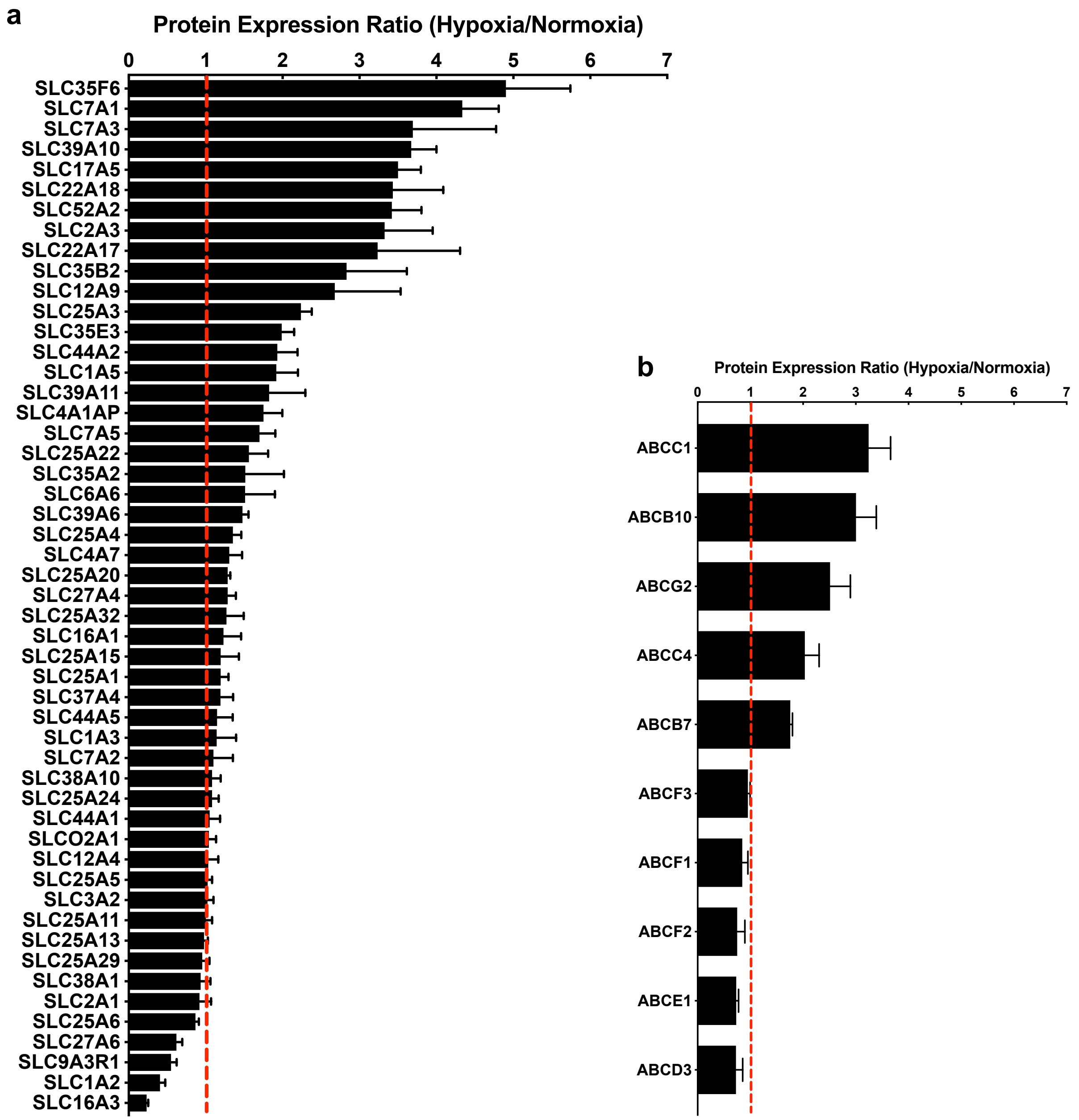

Supplementary Figure 3

All SLC proteins **(a)** and all ABC proteins **(b)** identified in the proteomics studies on the iPS-BMVEC differentiated under hypoxia and normoxia. Graphics show the relative abundance of the SLC and ABC proteins in the hBMVECs induced hypoxia vs normoxia conditions.

Supplementary Table 4: Raw proteomics data that used for generating Supplementary Fig. S3

Proteins	Protein Abundance				Protein Abundance Ratio	
	Normoxia		Hypoxia		Hypoxia/Normoxia	
	Average (N=3)	SE	Average (N=3)	SE	Average (N=3)	SE
**ABCC1**	1028.6	319.6	3419.8	1333.8	3.2	0.42
**ABCB10**	177.9	34.0	528.0	122.0	3.0	0.39
**ABCG2**	1478.4	533.7	3963.9	1788.1	2.5	0.38
**ABCC4**	152.0	99.1	260.7	141.4	2.0	0.27
**ABCB7**	1016.3	79.0	1782.9	141.2	1.8	0.04
**ABCF3**	794.0	248.7	737.9	216.3	1.0	0.04
**ABCF1**	3783.7	1527.5	2879.9	1038.3	0.8	0.11
**ABCF2**	2808.0	1372.2	1714.9	777.1	0.8	0.14
**ABCE1**	4564.1	1897.9	3200.7	1280.9	0.7	0.04
**ABCD3**	857.9	435.7	517.0	229.1	0.7	0.13
**SLC35F6**	163.6	16.7	777.7	69.9	4.9	0.84
**SLC7A1**	367.6	29.1	1566.8	57.3	4.3	0.47
**SLC7A3**	190.8	116.1	587.1	332.0	3.7	1.08
**SLC39A10**	426.9	97.9	1510.2	251.0	3.7	0.33
**SLC17A5**	221.3	99.1	726.7	308.7	3.5	0.30
**SLC22A18**	233.6	51.1	736.4	9.7	3.4	0.66
**SLC52A2**	189.2	30.4	663.9	153.2	3.4	0.38
**SLC2A3**	7358.9	1745.6	22427.4	1198.5	3.3	0.63
**SLC22A17**	221.9	62.7	713.6	240.6	3.2	1.07
**SLC35B2**	639.4	341.3	1401.5	456.1	2.8	0.78
**SLC12A9**	64.6	10.5	157.7	30.1	2.7	0.85
**SLC25A3**	11997.0	3795.8	27811.1	9513.2	2.2	0.14
**SLC35E3**	178.9	8.1	355.8	31.0	2.0	0.16
**SLC44A2**	552.7	165.9	1120.6	381.7	1.9	0.26
**SLC1A5**	1457.0	198.4	2701.1	224.3	1.9	0.28
**SLC39A11**	254.5	127.0	560.7	276.7	1.8	0.47
**SLC4A1AP**	132.5	48.7	255.3	105.1	1.8	0.24
**SLC7A5**	1473.1	219.1	2417.5	72.7	1.7	0.21
**SLC25A22**	627.4	69.0	992.3	200.4	1.6	0.25
**SLC35A2**	108.3	36.0	127.9	15.5	1.5	0.50
**SLC6A6**	325.6	68.8	539.7	196.2	1.5	0.38
**SLC39A6**	103.3	42.4	151.9	61.3	1.5	0.08
**SLC25A4**	966.6	434.5	1237.6	528.6	1.4	0.11
**SLC4A7**	152.5	48.2	199.7	72.8	1.3	0.17
**SLC25A20**	287.6	130.1	376.8	171.3	1.3	0.03
**SLC27A4**	306.5	110.5	404.0	149.2	1.3	0.11
**SLC25A32**	68.6	8.6	89.9	22.1	1.3	0.23
**SLC16A1**	2008.6	970.1	2091.5	650.1	1.2	0.23
**SLC25A15**	141.9	54.9	163.5	54.7	1.2	0.23
**SLC25A1**	1274.7	358.3	1580.8	500.1	1.2	0.10
**SLC37A4**	216.2	102.8	290.8	140.6	1.2	0.16
**SLC44A5**	567.5	394.9	794.0	631.6	1.1	0.20
**SLC1A3**	335.7	129.3	353.6	135.4	1.1	0.25
**SLC7A2**	252.9	94.3	316.3	135.4	1.1	0.25
**SLC38A10**	1895.2	802.5	2029.2	872.0	1.1	0.11
**SLC25A24**	4195.7	1797.1	4266.4	1732.7	1.1	0.09
**SLC44A1**	426.5	29.5	441.5	30.5	1.1	0.14
**SLCO2A1**	309.0	28.9	315.6	0.9	1.0	0.09
**SLC12A4**	346.4	174.4	316.6	150.3	1.0	0.13
**SLC25A5**	3166.1	1155.2	3126.7	1075.7	1.0	0.06
**SLC3A2**	6267.3	2277.3	6369.5	2238.3	1.0	0.08
**SLC25A11**	4954.4	858.7	5037.5	1032.7	1.0	0.08
**SLC25A13**	5998.5	2787.1	5956.6	2752.7	1.0	0.05
**SLC25A29**	86.7	31.8	80.1	26.5	1.0	0.09
**SLC38A1**	1895.2	802.5	2029.2	872.0	1.1	0.11
**SLC2A1**	2128.6	912.9	1759.8	595.2	0.9	0.15
**SLC25A6**	910.7	424.6	825.0	388.0	0.9	0.04
**SLC27A6**	201.3	69.3	114.2	33.0	0.6	0.07
**SLC9A3R1**	5058.6	1987.7	2639.4	903.2	0.5	0.07
**SLC1A2**	848.5	805.6	410.7	396.1	0.4	0.07
**SLC16A3**	657.1	154.1	158.9	52.1	0.2	0.02

Supplementary Table 5: Proteomics data for expression of ECM proteins in the iPS-BMVECs differentiated under hypoxia vs normoxia conditions

ECM Proteins	Name	Description	Protein Abundance				Protein Abundance Ratio		P Values
			Normoxia		Hypoxia		Hypoxia/Normoxia		
			Average (N=3)	SE	Average (N=3)	SE	Average (N=3)	SE	
Perlecan	HSPG2	Basement membrane-specific heparan sulfate proteoglycan core protein	7528.1	1833.5	20772.9	5309.5	2.8	0.8	0.004
Laminin	LAMA5	Laminin subunit alpha-5	4040.9	122.5	6473.7	159.8	1.6	0.0	<0.001
	LAMC1	Laminin subunit gamma-1	6503.2	3409.9	5865.8	2434.8	1.0	0.1	0.886
	LAMB1	Laminin subunit beta-1	7038.2	1703.3	6364.2	1330.1	0.9	0.0	0.771
Collagen IV	COL4A2	Collagen alpha-2(IV) chain	1850.3	947.4	4078.7	2358.1	2.1	0.2	0.430
	COL4A5	Collagen alpha-5(IV) chain	81.1	4.7	140.8	11.5	1.7	0.0	0.009
	COL4A6	Collagen alpha-6(IV) chain	263.9	129.7	300.9	125.4	1.2	0.2	0.847
Fibronectin	FN1	Fibronectin	85704.6	42217.2	90391.1	42530.3	1.1	0.1	0.941
SPARC	SPARC	SPARC	176.7	12.3	315.8	39.5	1.8	0.3	0.028
Agrin	AGRN	Agrin	2082.2	517.9	4049.0	970.5	2.0	0.2	0.148

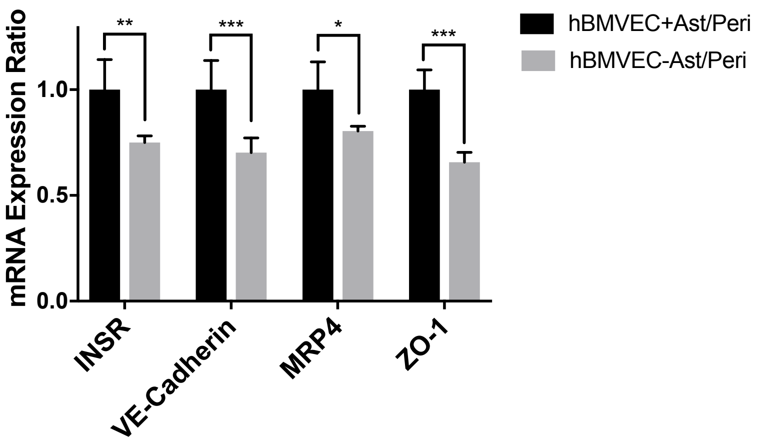

Supplementary Figure 4

mRNA expressions of INSR (insulin receptor protein), VE-cadherin, MRP4, and ZO-1 on the BBB Chips in the presence and absence of astrocyte and pericyte coculture were quantified using qPCR.

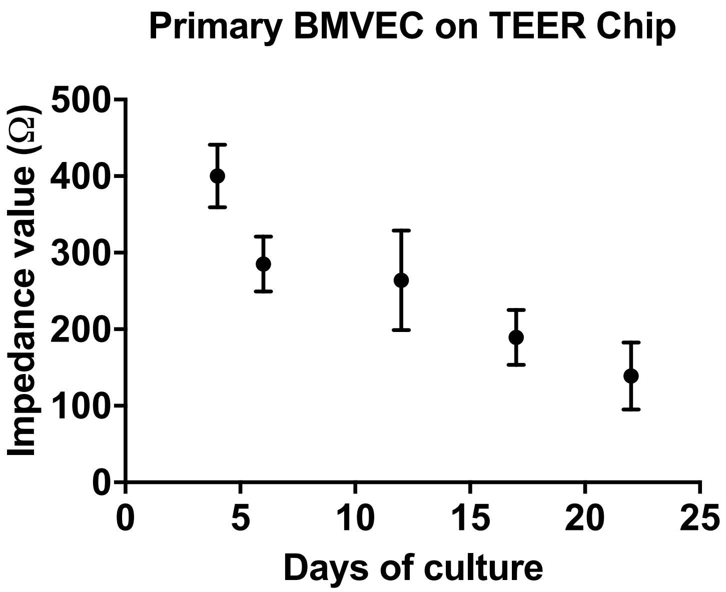

Supplementary Figure 5

Barrier integrity of the primary human BBB Chip monitored in TEER chips with impedance measurements, recorded in the frequency range of 0.1 Hz to 100 kHz over 3 weeks after seeding primary BMVECs along with astrocytes and pericytes.

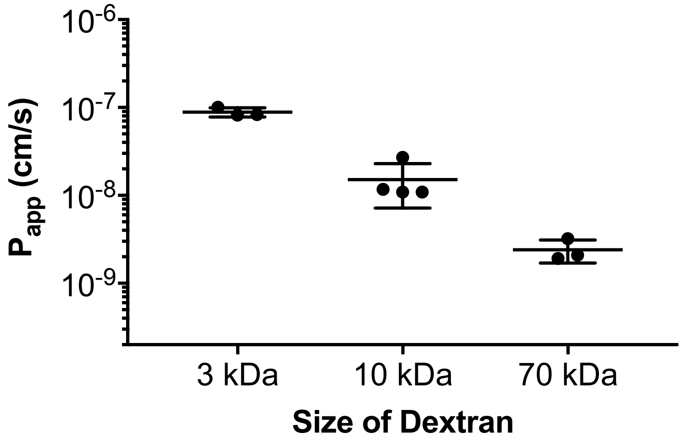

Supplementary Figure 6

Permeability of dextran tracers of various sizes in the BBB Chips. Fluorophore-labelled dextran molecules (3, 10, or 70 kDa) were flowed through the brain channel on the BBB Chips for 3 h at 100 μL/h flow rate. Effluent samples from both brain and vascular channels were collected and fluorescent intensity of the samples were detected, and dextran concentrations were quantified based on standard curves to calculate P_{app} values.

a

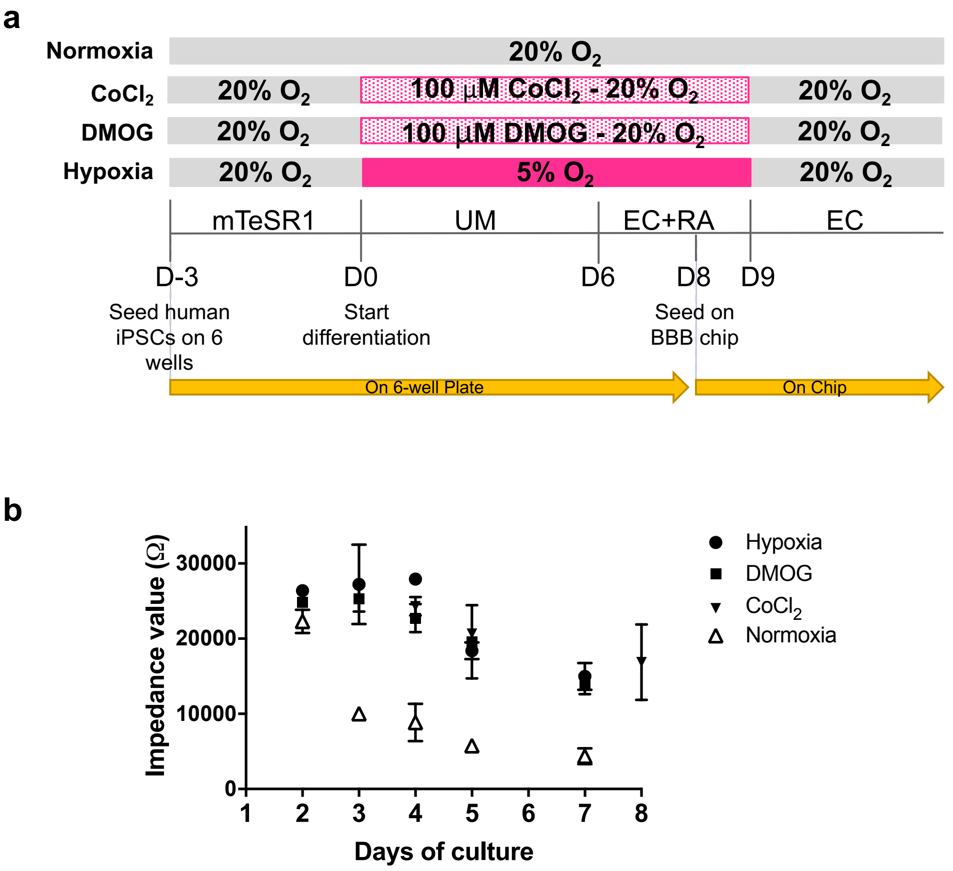

Supplementary Figure 7

(a) Experimental design schematic showing how BBB Chips were generated using iPS-BMVECs differentiated under normoxia (as control), hypoxia, or using chemical inducers ($CoCl_2$ and DMOG) that mimic hypoxia under normoxic conditions. **(b)** Impedance measurements of barrier integrity of BBB Chips generated with iPS-BMVECs differentiated as described in **a** and measured in TEER chips.

a

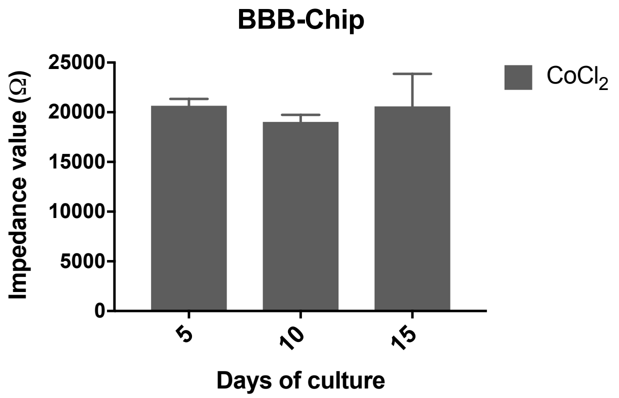

b

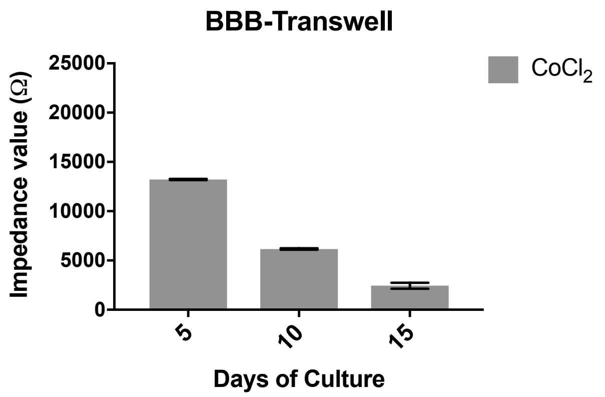

Supplementary Figure 8

The high barrier function (impedance measured by TEER) was sustained for longer times in BBB Chips with iPS-BMVECs that were induced to differentiate using $CoCl_2$ interfaced with astrocytes and pericytes(**a**) and this effect appeared to require flow as it was not observed when the same cells were interfaced in static Transwell cultures **(b).** Barrier integrity on the BBB Transwells was monitored by TEER measurements.

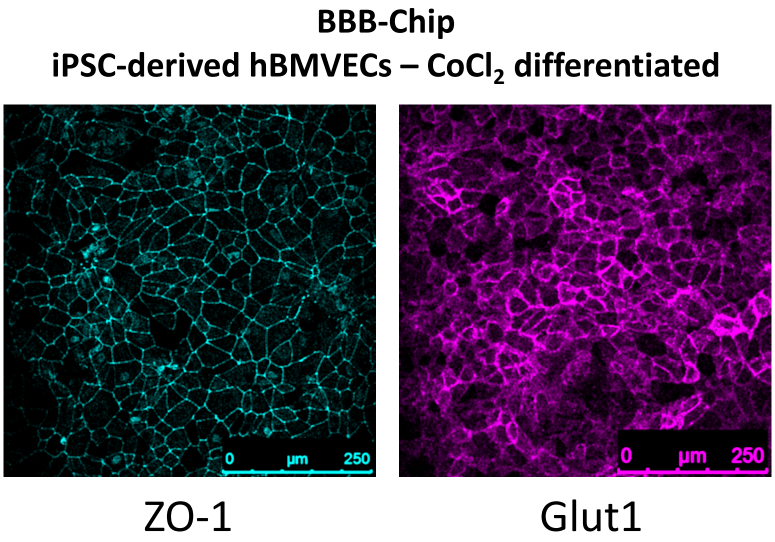

Supplementary Figure 9

Immunofluorescence micrographs showing the distribution of tight junction protein, ZO-1, (left) and glucose transfer protein, Glut1, (right) on the surface of the endothelium within BBB chips generated by using iPS-BMVECs differentiated in the presence of $CoCl_2$.

Supplementary Table 6: Papp values that used for generating Figure 3a and Figure 3b

BBB Chip						
**Pgp Inhibitor**	**Rhodamine**		**DiOC2**		**Citalopram**	
	Average	STD	Average	STD	Average	STD
-	1.29E-07	1.62E-09	2.36E-07	1.12E-08	7.33E-06	2.82E-06
+	2.86E-07	5.37E-08	3.30E-07	1.87E-08	2.29E-05	3.57E-07
**BBB Transwell**						
**Pgp Inhibitor**	**Rhodamine**		**DiOC2**		**Citalopram**	
	Average	STD	Average	STD	Average	STD
-	3.24E-07	4.11E-08	5.38E-07	1.38E-07	5.24E-05	5.43E-06
+	4.30E-07	2.36E-08	7.02E-07	2.73E-08	5.31E-05	7.74E-06

Supplementary Table 7: Papp values that used for generating Figure 3c

Rhodamine 123				
	Normoxia		Hypoxia	
	Average	STD	Average	STD
**Mock**	1.30E-07	1.74E-08	1.29E-07	1.62E-09
**Verapamil (Pgp)**	1.84E-07	3.04E-08	2.86E-07	5.37E-08
**MK571 (MRP1)**	2.16E-07	1.41E-08	2.00E-07	3.12E-08
**KO123 (BCRP)**	1.03E-07	2.13E-08	1.50E-07	1.22E-09
**DiOC2**				
	Normoxia		Hypoxia	
	Average	STD	Average	STD
**Mock**	2.36E-07	4.57E-08	2.36E-07	1.12E-08
**Verapamil (Pgp)**	2.39E-07	1.15E-08	3.30E-07	1.87E-08
**MK571 (MRP1)**	2.00E-07	6.41E-08	2.29E-07	1.52E-08
**KO123 (BCRP)**	2.18E-07	4.55E-08	3.85E-07	2.34E-09
**Doxorubicin**				
	Normoxia		Hypoxia	
	Average	STD	Average	STD
**Mock**	1.44E-06	2.25E-07	8.67E-07	1.82E-08
**Verapamil (Pgp)**	1.81E-06	2.40E-07	2.24E-06	2.57E-07
**MK571 (MRP1)**	1.46E-06	1.58E-07	8.22E-07	5.02E-08
**KO123 (BCRP)**	1.54E-06	1.37E-07	7.73E-07	3.62E-08

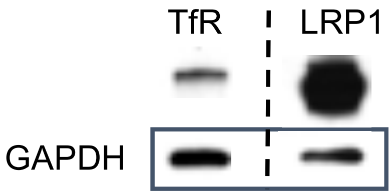

Supplementary Figure 10 Western Blot analysis of TfR and LRP1 protein expression on the iPS-BMVECs differentiated under hypoxic conditions. GAPDH as used as a control protein.

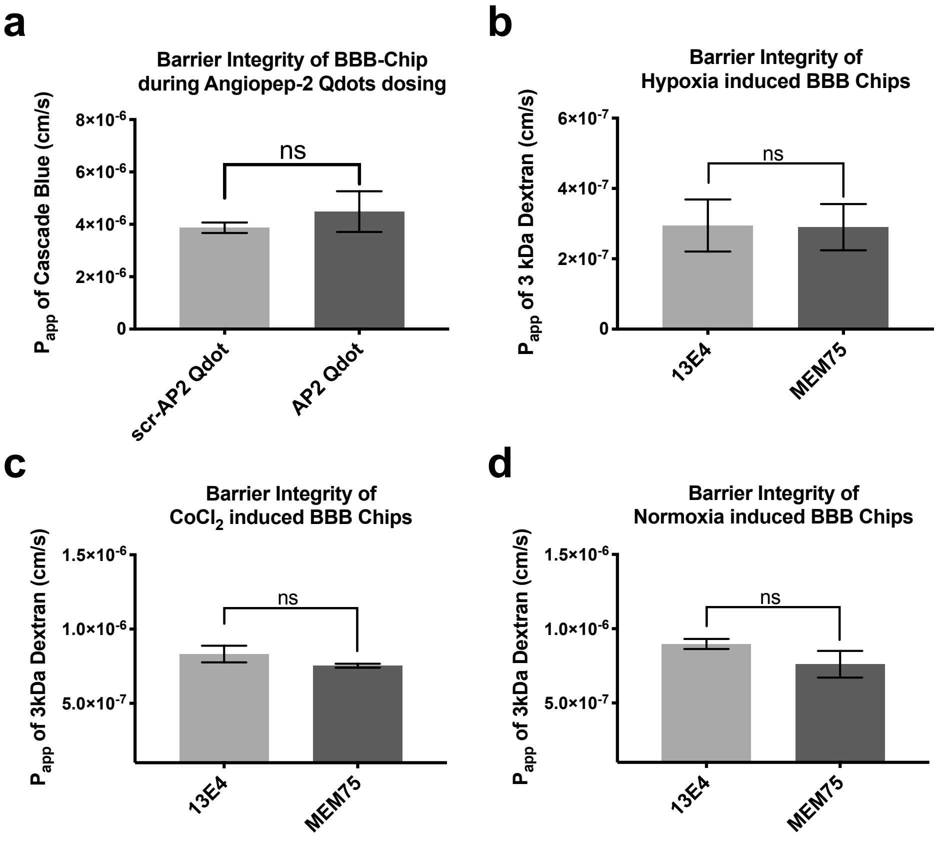

Supplementary Figure 11

No statistically significant differences (ns) in barrier integrity were detected in the BBB Chips in experiments measuring transcytosis of Angiopep-2 **(a)** or anti-TfR antibodies (MEM75 and 13E4) in iPS-BMVECs induced by hypoxia **(b)**, $CoCl_2$ **(c)**, or normoxia **(d)** as monitored by measuring the permeability of cascade blue (600 kDa), and 3 or 10 kDa dextran tracers.

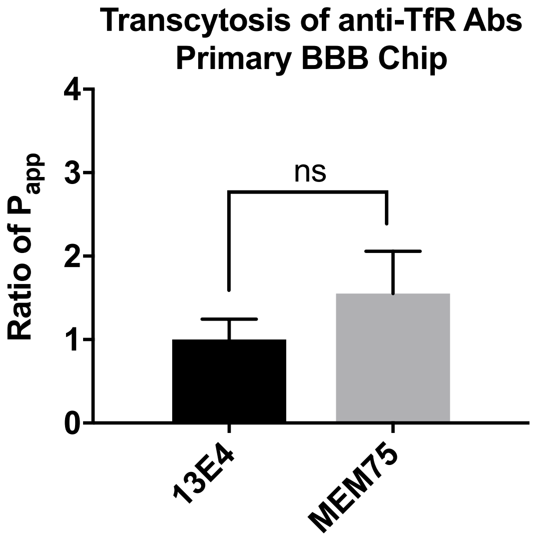

Supplementary Figure 12

Transcytosis of anti-TfR antibodies, MEM75 and 13E4, measured by quantifying their relative apparent permeability (Ratio of P_{app}) in the primary BBB Chip, demonstrating that there was no significant difference (N.S.) between the transcytosis abilities of the two anti-TfR antibodies when primary human brain endothelial cells were used instead of iPS-BMVECs in the BBB Chip.

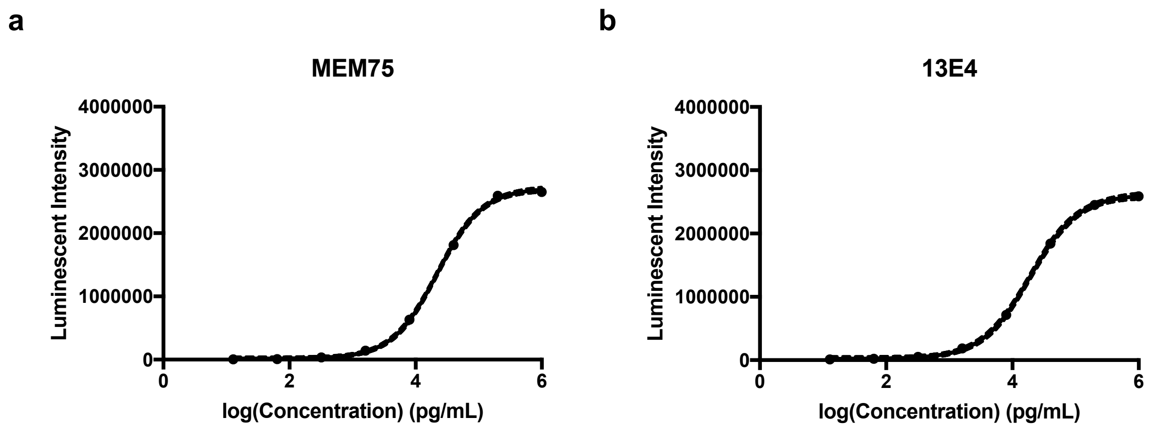

Supplementary Figure 13

Standard curves for the binding of **(a)** MEM75 and **(b)** 13E4 antibodies to iPS-BMVECs that were used in the ELISA experiment.
