# Supplementary material for: Hypoxia-enhanced Blood-Brain Barrier Chip recapitulates human barrier function and shuttling of drugs and antibodies
Source: Nat Commun. 2019 Jun 13;10:2621. doi: 10.1038/s41467-019-10588-0 (PMC6565686; doi:10.1038/s41467-019-10588-0)
Supplement: Supplementary file 3 — Description of Additional Supplementary Files [file 41467_2019_10588_MOESM3_ESM.pdf]

Description of Additional Supplementary Files

File name: Supplementary Movie 1

Description: The 3D BBB Chip model containing human iPS-BMVECs (ZO1, blue and DAPI, purple) cultured on all four sides of the bottom channel, with human primary astrocytes (GFAP, white) and human primary pericytes (F-actin, yellow) co-cultured on the upper surface of the matrix-coated porous membrane in the top channel.

File name: Supplementary Movie 2

Description: Higher magnification views of z-stack images showing astrocytes on the upper surface of the matrix-coated porous membrane extending membrane processes (GFAP, purple) through the pores of the membrane to make direct contacts with the endothelial cells on its lower surface in the bottom channel.
