# Supplementary material for: Hypoxia-enhanced Blood-Brain Barrier Chip recapitulates human barrier function and shuttling of drugs and antibodies
Source: Nat Commun. 2019 Jun 13;10:2621. doi: 10.1038/s41467-019-10588-0 (PMC6565686; doi:10.1038/s41467-019-10588-0)
Supplement: Supplementary file 6 — Reporting Summary [file 41467_2019_10588_MOESM6_ESM.pdf]

nature research

Corresponding author(s): Donald E. Ingber

Reporting Summary

Nature Research wishes to improve the reproducibility of the work that we publish. This form provides structure for consistency and transparency in reporting. For further information on Nature Research policies, see Authors & Referees and the Editorial Policy Checklist.

Statistical parameters

When statistical analyses are reported, confirm that the following items are present in the relevant location (e.g. figure legend, table legend, main text, or Methods section).

n/a	Confirmed	
☐	☒	The exact sample size (*n*) for each experimental group/condition, given as a discrete number and unit of measurement
☐	☒	An indication of whether measurements were taken from distinct samples or whether the same sample was measured repeatedly
☐	☒	The statistical test(s) used AND whether they are one- or two-sided<br>*Only common tests should be described solely by name; describe more complex techniques in the Methods section.*
☐	☒	A description of all covariates tested
☐	☒	A description of any assumptions or corrections, such as tests of normality and adjustment for multiple comparisons
☐	☒	A full description of the statistics including central tendency (e.g. means) or other basic estimates (e.g. regression coefficient) AND variation (e.g. standard deviation) or associated estimates of uncertainty (e.g. confidence intervals)
☐	☒	For null hypothesis testing, the test statistic (e.g. *F*, *t*, *r*) with confidence intervals, effect sizes, degrees of freedom and *P* value noted<br>*Give P values as exact values whenever suitable.*
☒	☐	For Bayesian analysis, information on the choice of priors and Markov chain Monte Carlo settings
☐	☒	For hierarchical and complex designs, identification of the appropriate level for tests and full reporting of outcomes
☒	☐	Estimates of effect sizes (e.g. Cohen's *d*, Pearson's *r*), indicating how they were calculated
☐	☒	Clearly defined error bars<br>*State explicitly what error bars represent (e.g. SD, SE, CI)*

Our web collection on statistics for biologists may be useful.

Software and code

Policy information about availability of computer code

Data collection	BioTek plate reader software: Synergy Neo GEN5 2.09<br>Metrohm Autolab BV: Nova Software<br>QuantStudio(TM) 7 Flex System (Serial # 278870290) Software V1.0<br>Proteomics mass spec data software: XCalibur 3.0<br>Zeiss Software: ZEN2 Blue edition 2.0.14283.302<br>Olympus Software: Metamorph Premier7.8.12.0
Data analysis	Microsoft Excel 365 and Prism 7 was used for statistical analysis.<br>Proteomics data: Proteome Discoverer 1.4 (Thermo Scientific) software. Assignment of MS/MS spectra was performed using the Sequest HT algorithm by searching the data against the Uniprot_HUMAN database.

For manuscripts utilizing custom algorithms or software that are central to the research but not yet described in published literature, software must be made available to editors/reviewers upon request. We strongly encourage code deposition in a community repository (e.g. GitHub). See the Nature Research guidelines for submitting code & software for further information.

Data

Policy information about availability of data

All manuscripts must include a data availability statement. This statement should provide the following information, where applicable:

- Accession codes, unique identifiers, or web links for publicly available datasets
- A list of figures that have associated raw data
- A description of any restrictions on data availability

In the Manuscript the following is stated:
Data Availability. All data generated or analysed during this study are included in this published article (and its supplementary information files), and all raw data will be available upon request.

Field-specific reporting

Please select the best fit for your research. If you are not sure, read the appropriate sections before making your selection.

☒ Life sciences ☐ Behavioural & social sciences ☐ Ecological, evolutionary & environmental sciences

For a reference copy of the document with all sections, see nature.com/authors/policies/ReportingSummary-flat.pdf

Life sciences study design

All studies must disclose on these points even when the disclosure is negative.

Sample size	Samples size was equal or larger than 3 in all experiments. No method was used to calculate the sample size.
Data exclusions	Data was not excluded from the experiment unless apparent failures due to following reasons:<br>1- Hardware failure (e.g. pump error, tubing unplugging),<br>2- Human errors for setting up the experiments<br>3- Contamination problems
Replication	Experiments were repeated with several chip set-ups and on multiple occasions.
Randomization	All chips were randomly assigned for antibody or peptide or isotonic buffer treatments and for controls
Blinding	The investigators were not blind to the design of the study; however, antibody quantification and proteomics studies were carried out blinded by the operators.

Reporting for specific materials, systems and methods

Materials & experimental systems

n/a	Involved in the study
☐	☒ Unique biological materials
☐	☒ Antibodies
☒	☐ Eukaryotic cell lines
☒	☐ Palaeontology
☒	☐ Animals and other organisms
☒	☐ Human research participants

Methods

n/a	Involved in the study
☒	☐ ChIP-seq
☒	☐ Flow cytometry
☒	☐ MRI-based neuroimaging

Unique biological materials

Policy information about availability of materials

Obtaining unique materials	All biological materials were obtained from commercial sources:<br>1- iPSC IMR90-C4, WiCell Research Institute, Cat#: WISCi004-B<br>2- Human Astrocytes, ScienCell, Cat#:1800<br>3- Human brain varcular pericytes, ScienCell, Cat#: 1200

Antibodies

Antibodies used

1- Goat anti-mouse IgG, Fc-gamma specific, Jackson ImmunoResearch, Cat#: 115-005-164, Lot#: 125566
2- Claudin-5, host: mouse, target: human, conjugation: Alexa Fluor 488, Invitrogen, Cat#: 352588, Lot#: RD234858
3- VE-Cadherin, Clone: BV9, Host: Mouse, Target: Human, conjugation: Alexa Fluor 647, BioLegend, Cat#: 348514, Lot#: B183851
4- anti-CD31 (PECAM-1), Clone: WM59, Host: Mouse, Target: Human, conjugation: Alexa Fluor 594, Biolegend, Cat#: 303126, Lot#: B199682
5- anti-ZO1, Clone: ZO1-A12, Host: Mouse, Target: Human, Dog, Conjugate: Alexa Fluor 594, Invitrogen, Cat#: 339194, Lot#: RA222626
6-anti-Pgp, Clone: EPR10365-57, Host: Rabbit, Target: Human, Abcam, Cat#: ab170904, Lot#: GR217576-14
7-anti-GLUT1, Clone: EPR3915, Host: Rabbit, Target: Human, Conjugation: Alexa Fluor 488, Abcam, Cat#: ab195359, Lot#: GR3187483-13
8-anti-GFAP, Clone: EPR1034Y, Host: Rabbit, Target: Human, Rat, Conjugation: Alexa Fluor 647, Abcam, Cat#: ab194325, Lot#: GR219425-2
9-anti-alpha smooth muscle actin, Clone: EPR5368, Host: Rabbit, Target: Human, Rat, Conjugation: Alexa Fluor 555, Abcam, Cat#: ab202509, Lot#: GRGR3194363-1
10-GAPDH, Clone: D16H11-XP, Host: Rabbit, Target: Human, Cell Signaling Technology, Cat#: 5174
11-Cetuximab, Human IgG1, Source: CHO Cells, Selleckchem, Cat #: A2000, Pharmaceutical Antibody
12-anti-Transferrin Receptor Antibody, Clone: 13E4, Host: Mouse, Target: Human, Abcam, Cat#: ab38171
13-anti-Transferrin Receptor Antibody, Clone: MEM75, Host: Mouse, Target: Human, Abcam, Cat#: ab9179
14-Goat anti-mouse IgG, F(ab')2 specific, Conjugate: HRP, Jackson ImmunoResearch, Cat#: 115-035-072, Lot#: 134947
15-Goat anti-rabbit IgG, (H+L), Conjugate: HRP, Jackson ImmunoResearch, Cat#: 115-035-045, Lot#: 137251
16-anti-LRP1 Antibody, Clone EPR3724, Host: Rabbit, Target: Mouse, Rat, Human, Pig, Abcam, Cat#: ab92544

Validation

All validation statements were taken from the suppliers website.
1- Based on immunoelectrophoresis and/or ELISA, the antibody reacts with mouse IgG subclasses 1, 2a, 2b, and 3; but not with the Fab portion of mouse immunoglobulins. No antibody was detected against mouse IgM or non-immunoglobulin serum proteins. The antibody has been tested by ELISA and/or solid-phase adsorbed to ensure minimal cross-reaction with human, bovine and rabbit serum proteins, but it may cross-react with immunoglobulins from other species
2- Reactivity has been confirmed with rat, human and mouse Claudin-5 using rat lung, mouse kidney, mouse small intestine, mouse lung homogenates, human colon tissue, and CACO-2 human cell line. This antibody reacts specifically with the ~ 22-24 kDa endogenous Claudin-5 protein.
3- Each lot of this antibody is quality control tested by immunofluorescent staining with flow cytometric analysis. For flow cytometric staining, the suggested use of this reagent is 5 µl per million cells in 100 µl staining volume or 5 µl per 100 µl of whole blood. For immunohistochemical staining on formalin-fixed paraffin-embedded tissue sections, a concentration range of 5 - 10 µg/ml is suggested. It is recommended that the reagent be titrated for optimal performance for each application.
4- Each lot of this antibody is quality control tested by immunofluorescence staining. For immunofluorescence microscopy, a concentration range of 5-10 µg/ml is recommended. It is recommended that the reagent be titrated for optimal performance for each application.
5- This antibody was tested with the applications of ELISA, immunocytochemistry, immunoflurescence, western blot; and used in publications for the applications of immunofluorescence, immunoscytochemistry, and others.
6- ab170904 detects a predominant protein band migrating in the region of 180-200 kDa and typically will demonstrate a smear on the membrane in the region of the 150 – 300 kDa due to the glycosylation profile of the protein.
7- Abpromise guarantee covers the use of ab195359 in the following tested applications: immunocytochemistry, immunoflurescence, and Flow Cytometry
8- Abpromise guarantee covers the use of ab194325 in the following tested applications: IHC/Fr
9- Abpromise guarantee covers the use of ab195359 in the following tested applications: immunocytochemistry, immunoflurescence. Positive control: ICC/IF on SV40LT-SMC cells.
10- Monoclonal antibody is produced by immunazing animals with a synthetic peptide corresponding to residues near the carboxy terminus of human GAPDH. Species Reactivity: Human, Mouse, Rat, Monkey. Applications: Western Blot, IHC-P, ICC/IF
11- Cetuximab is a recombinant chimeric monoclonal antibody that binds to the human epidermal growth factor receptor (EGFR) with high affinity. Binding to EGFR blocks phosphorylation and activation of receptor-associated kinases which results in cell growth inhibition, induction of apoptosis, and decreased vascular endothelial growth factor production.
12- This antibody is specific for Transferrin Receptor and the complex between soluble Transferrin Receptor and Transferrin. This antibody gave a positive result when used in the following formaldehyde fixed cell lines: DU145. Suitable for: ELISA, WB, ICC/IF, Flow Cytometer.
13- This antibody does not block the binding of transferrin to the receptor. This antibody gave a positive result in IF in the following Formaldehyde fixed cell line: DU145. Suitable for: ICC/IF, Flow Cyt, IP.
14- Based on immunoelectrophoresis and/or ELISA, the antibody reacts with the F(ab')2/Fab portion of mouse IgG. It also reacts with the light chains of other mouse immunoglobulins. No antibody was detected against the Fc portion of mouse IgG or against non-immunoglobulin serum proteins. The antibody has been tested by ELISA and/or solid-phase adsorbed to ensure minimal cross-reaction with human, bovine and horse serum proteins, but it may cross-react with immunoglobulins from other species.
15- Based on immunoelectrophoresis and/or ELISA, the antibody reacts with whole molecule mouse IgG. It also reacts with the light chains of other mouse immunoglobulins. No antibody was detected against non-immunoglobulin serum proteins. The antibody may cross-react with immunoglobulins from other species.
16- WB: PMBC and A549 cell lysates; mouse brain, heart, kidney and spleen tissue lysates; rat brain, heart, kidney or spleen tissue lysates; human fetal brain tissue lysates; pig liver and heart tissue lysates. IHC-P: Human liver, clear cell carcinoma, brain, lung and placenta tissues. ICC/IF: U87-MG cells. Flow Cyt: Jurkat cells. IP: A549 cells.
